# Editorial: Cross-reactive immunity and COVID-19

**DOI:** 10.3389/fimmu.2024.1509379

**Published:** 2024-12-09

**Authors:** Aristo Vojdani, Ahmed Yaqinuddin, Alberto Beretta, Pedro A. Reche

**Affiliations:** ^1^ Laboratory, Immunosciences Lab., Inc., Los Angeles, CA, United States; ^2^ Administration, Cyrex Labs, LLC, Phoenix, AZ, United States; ^3^ Research and Graduate Studies, Graduate Programs, College of Medicine, Alfaisal University, Riyadh, Saudi Arabia; ^4^ Solongevity Research, SoLongevity Healthcenters, Milano, Italy; ^5^ Dpto de Immunologia, Facultad de Medicina, U. Complutense de Madrid, Madrid, Spain

**Keywords:** cross-reactivity, COVID-19, long COVID, SARS-CoV-2, vaccines, pre-existing immunity

SARS-CoV-2 and COVID-19 impacted the world like a modern plague, stretching icy fingers across the globe. Humanity was even more horrified upon finding that this new plague came with variants, and surviving one version of the virus did not automatically grant immunity to all variants. But as has happened with previous pandemics, the deadliness and reach of COVID-19 has subsided into a slightly worrisome status quo due to the advancements of medical science and the virus’ own evolution into less harmful forms. The intense scrutiny that SARS-CoV-2 and COVID-19 received during the height of their deadly assault on the world has revealed things that are now used to combat this pandemic.

**Figure 1:**
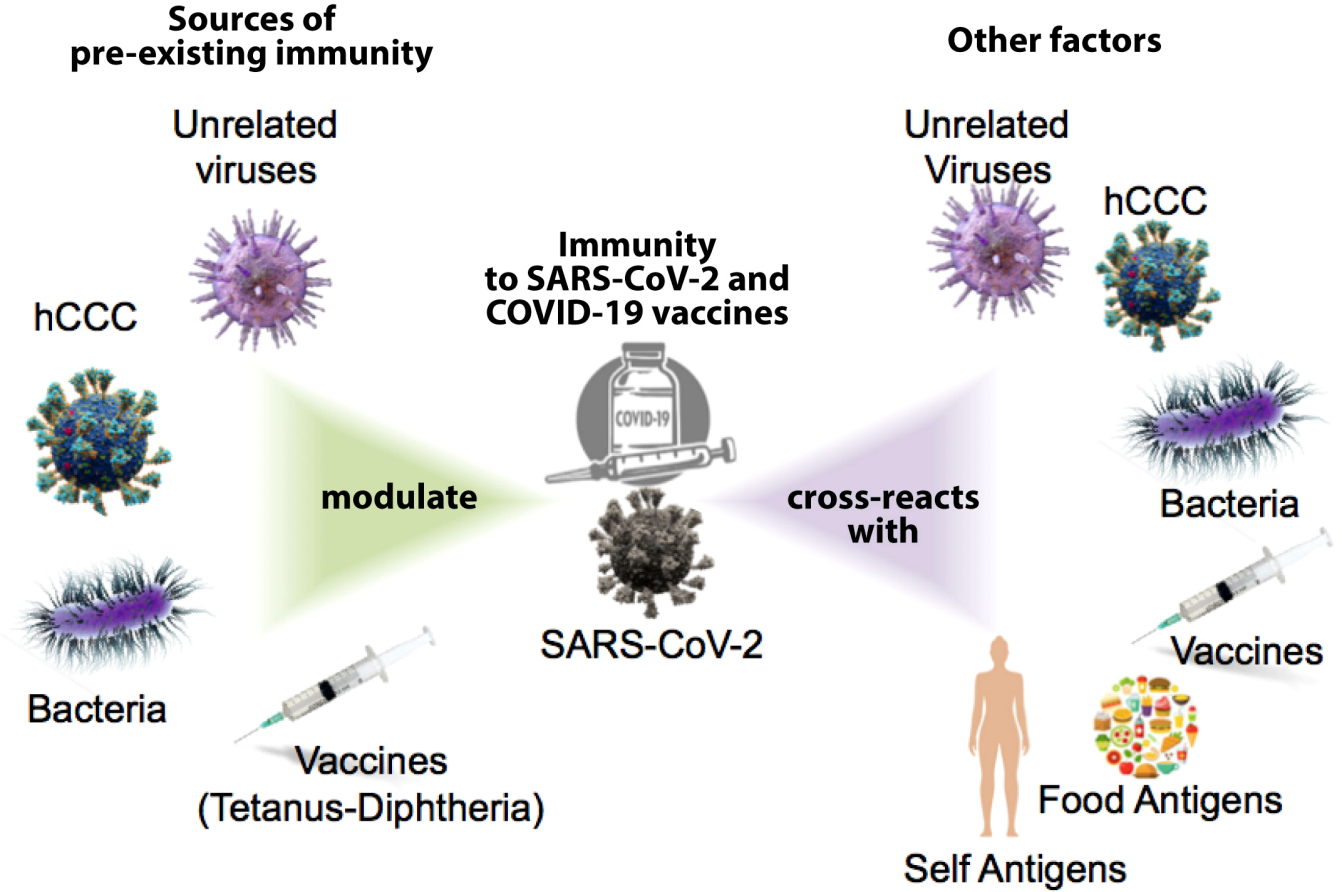
Potential sources of present immunity shape the response to SARS-CoV-2 and the COVID-19 vaccine; the immune response to SARS-CoV-2 and COVID vaccines can also produce cross-reactive immune responses to other factors.

We now know that, unlike us, our immune system was not so surprised by SARS-CoV-2 since cross-reactive immunity to SARS-CoV-2 existed prior to the COVID-19 pandemic. Cross-reactive immunity is mediated by antibodies and memory B and T cells elicited by a specific pathogen or antigen that can also react to other pathogens or antigens (1) Cross-reactivity is a main feature of adaptive immunity, which is highly favored by the recognition of small portions within protein antigens (epitopes) (2) and the poly-specificity of cognate B and T cell receptors (3, 4). Human common cold coronaviruses (hCoVs) have received major attention as potential sources of cross-reactive immunity to SARS-CoV-2 (5). However, immune cross-reactivity has also been reported between SARS-CoV-2 and unrelated viruses (6), bacteria (7), vaccines (8, 9) and even food antigens (9). Activation of cross-reactive immunity is not always protective and can also produce immunopathology (10). Moreover, immune cross-reactivity is a two-way road and SARS-CoV-2 infection as well as COVID-19 vaccines can also induce cross-reactive immunity. Indeed, immune cross-reactivity between SARS-CoV-2 and COVID-19 vaccines with human tissues has been shown, raising the possibility that autoimmune reactivity can result from SARS-CoV-2 infection and COVID-19 vaccines (see **Figure 1**) (11).

Clearly, pre-existing cross-reactive immunity must have a major impact in shaping the immune response to the virus and to COVID-19 vaccines, but to what extent and its contribution to protection is still undetermined. Likewise, SARS-CoV-2 and COVID-vaccines can have profound cross-reactive immunological consequences that need to be investigated. In this Research Topic of Frontiers in Immunology about “*Cross-Reactive Immunity and COVID-19*,” editors Aristo Vojdani, Pedro Reche, Alberto Beretta and Ahmed Yaqinuddin have gathered an impressive Research Topic of articles investigating sources of immune cross-reactivity with SARS-CoV-2, contribution to protection, COVID-19 course and its ancillary condition, long COVID.

On the face of it, it would seem that cross-reactive immunity would be a welcome weapon to wield against COVID-19. Pedro Reche, one of the editors of this Research Topic, published an article in 2020 (8) in which he concluded, “Overall, our results clearly support that cross-reactive immunity from DTP vaccines can be protecting children against SARS-CoV-2 and could protect the general population.” In 2022, Fonte et al. looked at the lower susceptibility of children to SARS-CoV-2 infection and concluded that the lower incidence and severity of the disease in children could be due to nonspecific resistance to SARS-CoV-2 generated by the childhood vaccines received by the subjects (12).

The value of this general protection or immunity against SARS-CoV-2 and its variants is echoed by Chao et al.‘s contribution to this Research Topic. These authors report that broadly neutralizing ability is critical for developing the next generation SARS-CoV-2 vaccine. They found that a specific homologous vaccine was insufficient for dealing with the Omicron virus variant, which had very different antigenic characteristics from the original. They also found that anti-ACE2 autoantibodies were significantly increased in all vaccinated test groups.

The Omicron variant also proved problematic for Li M. et al.‘s study in this Research Topic. They found that vaccination contributes significantly in limiting the spread of SARS-CoV-2. However, they also found that this vaccine protection is not efficient against the Omicron variant. This was evidenced by the reduction in the binding of SARS-CoV-2 antibody-positive human sera to Omicron RBDs compared to its homogenous recombinant RBDs.

In their own article, Lunderberg et al. had a slightly different suggestion for dealing with the problem of SARS-CoV-2 variants escaping the host’s immune response. Previously, researchers have identified IgG1 type pan-neutralizing antibodies (neutAbs) which can effectively neutralize several human coronaviruses, including SARS-CoV-2 and its variants, but with existing limitations and deficiencies in their efficiency and application. Lunderberg et al. proposed that using Reverse Technology 3.0 can help to further the use of this innate-like defense mechanism.

We have already mentioned that the emergence of SARS-CoV-2 variants prevents acquiring general immunity to the disease. Thompson et al. investigated the impact of cross-reactive immunity on the emergence of these variants. They found that if cross-reactivity immunity is complete, that the antigenically related novel variant must be more transmissible than the previous virus in order to invade the population. They highlighted that a fast assessment of the level of cross-reactive immunity conferred by related viruses against novel SAR-CoV-2 variants is essential to assessing the risk posed by those variants.

The more we know about SARS-CoV-2 variants, then, the better it is for understanding the virus, its diseases, and how to deal with them. Kuthning et al. monitored SARS-CoV-2 specific antibodies in children and adolescents to determine whether the S1-specific antibody response can identify the infecting variant of concern (VoC), estimate the prevalence of silent infections, and test whether vaccination or infection with SARS-CoV-2 induces hCoV cross-reactive antibodies. They concluded that the antibody response to the S1 domain of the SARS-CoV-2 spike protein is highly specific, providing important information about the infecting VoC and revealing clinically silent infections.

The source and role of SARS-CoV-2 cross-reactive antibodies have received major attention in this Research Topic. In an article by Peng et al., the authors show that monoclonal antibodies constructed from COVID-19 convalescent memory B cells exhibited potent binding activity to the S2 spike subunit from MERS-CoV and other hCoVs. The authors did not investigate whether the cross-reactive antibodies resulted from the activation of pre-existing memory B cells or from the activation of naive B cells but, regardless, their results will be useful in the diagnosis of multiple coronaviruses. Although the S2 spike subunit SARS-CoV-2 can be the target of neutralizing antibodies, most of them target the receptor binding domain (RBD) in the spike S1 subunit, which is much less conserved that the S2 subunit (13).

Consistently, Adami et al. report that anti-RBD IgG antibodies from endemic hCoVs do not protect against the acquisition of SARS-CoV-2 infection among exposed uninfected individuals. In line with this result, Lin et al. reports that pre-existing humoral immunity to hCoVs negatively impacts the protective SARS-CoV-2 antibody response (14). Additionally, in one of the articles published in this Research Topic, Li N et al. looked at whether pre-existing antibodies induced by low pathogenic human coronaviruses (LPH-CoVs) in children can cross-react with SARS-CoV-2. They found that the seroprevalence of four analyzed LPH-CoVs reached 75.84%, and about 24.64% of the seropositive samples had cross-reactive IgG antibodies against the nucleocapsid S and against the receptor binding domain (RBD) antigens of SARS-CoV-2. These data suggest that children’s pre-existing antibodies to LPH-CoVs have limited cross-reactive neutralizing antibodies against SARS-CoV-2.

In contrast, Cheng Y. et al. report antigenic cross-reactivity between the SARS-CoV-2 RBD and dengue virus, which in dengue patients can lead to anti-SARS-CoV-2 S1-RBD antibodies hindering pathogenicity. Likewise, SARS-CoV-2 and COVID-19 vaccines may in some occasions lead to pathological antibodies due to cross-reactivity. This possibility is illustrated by the case report presented by Shimizu et al., showing a new-onset dermatomyositis following COVID-19. The diagnosis was confirmed by the detection of anti-synthetase autoantibodies and other biochemical analyses. However, they did not provide evidence that this development of dermatomyositis may be due to cross-reactivity between SARS-CoV-2 antigens and tissue antigens involved in this disease. Zhong et al. present two case reports in which patients with bacterial pneumonia and high elevated immunoglobins did not get infected with SARS-CoV-2. This report provides experimental support onto the possibility that bacteria can elicit protective cross-reactive antibodies to SARS-CoV-2, as suggested by *in silico* works (15).

Unlike cross-reactive humoral immunity, which may enhance disease severity (16), there is mounting evidence of the protective role of SARS-CoV-2 cross-reactive T cells (17). The work by Coulon et al. support a protective role for alpha-hCoV-specific memory T cells cross-reactive with SARS-CoV-2. However, hCoV cannot account for all pre-existing SARS-CoV-2 cross-reactive T cells (18), and it is still unproven that hCoV can prime T cells cross-reactive with SARS-CoV-2. Interestingly, such a proof is provided in this Research Topic for tetanus-diphtheria vaccine. Thus, Fernandez et al. showed that antigen-inexperienced naive T cells primed with tetanus-diphtheria vaccine recognized SARS-CoV-2-specific CD8 T cell epitopes, as anticipated by Reche in a seminal in *silico* study (8). Given that children received several immunizations with vaccines containing tetanus-dihptheria antigens, the results by Fernandez et al. support that tetanus-diphtheria vaccine likely had a major contribution shaping exiting SARS-CoV-2 T cell responses. However, pre-existing cross-reative T cells may not always be protective. Thus, Ng’uni et al. report that pre-existing endemic NL63-coronavirus-specific T cells are associated with impaired SARS-CoV-2-specific T cell responses.

Continuing the thread of the protective or supportive role of T cells against SARS-CoV-2, in their article in this Research Topic, Ziehe et al. sought to study the human cytomegalovirus (HCMV) as a risk factor for the development of sepsis in COVID 19 patients. They found that although HCMV was significantly higher in COVID-19 patients compared to controls, the cross-reactivity of HCMV-specific CD8+ T cells with SARS-CoV-2 peptides might actually confer some protection to HCMV-seropositive patients.

On the other hand, Leung et al. found something interesting about B cells. We know that infection by SARS-CoV-2 can lead past COVID-19 to long COVID. Leung et al. examined different early immune factors in both hospitalized and non-hospitalized patients with COVID-19, and correlated the immune factors with the development of long COVID. They found that the predominant early immune indicator of long COVID was double-negative B cells, indicating a potentially important role for these cells in the development of the disease.

In another attempt to predict the progression and severity of COVID-19, You et al. measured serum levels of ACE2 and AXL in patients who were categorized into non-severe and severe cases. They compared these levels with SARS-CoV-2 IgG and IgM antibody titers at different time points in post-COVID infections. They found that in severe COVID-19 cases, a decrease in AXL level with an increase in SARS-CoV-2 IgG level predicts COVID-19 progression.

Pre-existing cross-reactive immunity have surely shaped the responses to COVID-19 vaccines. However, some COVID-19 vaccines may activate more than other cross-reactive immunity. This fact is exemplified in the report by Henze et al. These authors show that adenovirus-vector-based ChAdOx1 vaccine did not reactivate cross-reactive cellular and humoral immunity compared to mRNA-based BNT162b2.

One of this Research Topic’s submissions had suggestions for treatment. Saito et al. observed that a significant percentage of SARS-CoV-2-infected individuals develop long COVID which overlaps with myalgic encephalomyelitis/chronic fatigue syndrome (ME/CFS). They identified alterations in metabolic pathways, including the elevation of plasma pro-inflammatory cytokines with a reduction in ATP in long COVID patients. These metabolic abnormalities not only help in better understanding the pathophysiology of long COVID but also in finding supplements with potential therapeutic implications which were suggested by the researchers.

Taken all together, the 19 articles in this Research Topic examine cross-reactive immunity concerning SARS-CoV-2 and COVID-19. They highlight the intricate interplay between prior immune responses and the evolving viral landscape. The information obtained implies that response to antibodies and immune cells are likely to be critical in both the risk of infection and the response to vaccines. Although there are encouraging signs that pre-existing immunity may provide a degree of protection, the heterogeneity in responses—particularly against new variations such as Omicron—suggests that this phenomenon is not universally effective. As the pandemic evolved, it became evident that understanding of mechanisms involved in cross-reactive immunity will be essential for developing strategies to fight against COVID-19 and its variants.
